# Diet’s Impact on Post-Traumatic Brain Injury Depression: Exploring Neurodegeneration, Chronic Blood–Brain Barrier Destruction, and Glutamate Neurotoxicity Mechanisms

**DOI:** 10.3390/nu15214681

**Published:** 2023-11-04

**Authors:** Matthew Boyko, Benjamin F. Gruenbaum, Anna Oleshko, Igor Merzlikin, Alexander Zlotnik

**Affiliations:** 1 Department of Anesthesiology and Critical Care, Soroka University Medical Center, Ben-Gurion of the Negev, Beer-Sheva 84101, Israel; alekszl@clalit.org.il; 2 Department of Anesthesiology and Perioperative Medicine, Mayo Clinic, Jacksonville, FL 32224, USA; gruenbaum.benjamin@mayo.edu; 3 Department of Biology and Methods of Teaching Biology, A. S. Makarenko Sumy State Pedagogical University, Sumy 40002, Ukraine; annaoleshko8567@gmail.com (A.O.); mirdaodzi@gmail.com (I.M.)

**Keywords:** blood–brain barrier, depression, diet, glutamate, traumatic brain injury

## Abstract

Traumatic brain injury (TBI) has a profound impact on cognitive and mental functioning, leading to lifelong impairment and significantly diminishing the quality of life for affected individuals. A healthy blood–brain barrier (BBB) plays a crucial role in guarding the brain against elevated levels of blood glutamate, making its permeability a vital aspect of glutamate regulation within the brain. Studies have shown the efficacy of reducing excess glutamate in the brain as a treatment for post-TBI depression, anxiety, and aggression. The purpose of this article is to evaluate the involvement of dietary glutamate in the development of depression after TBI. We performed a literature search to examine the effects of diets abundant in glutamate, which are common in Asian populations, when compared to diets low in glutamate, which are prevalent in Europe and America. We specifically explored these effects in the context of chronic BBB damage after TBI, which may initiate neurodegeneration and subsequently have an impact on depression through the mechanism of chronic glutamate neurotoxicity. A glutamate-rich diet leads to increased blood glutamate levels when contrasted with a glutamate-poor diet. Within the context of chronic BBB disruption, elevated blood glutamate levels translate to heightened brain glutamate concentrations, thereby intensifying neurodegeneration due to glutamate neurotoxicity.

## 1. Introduction

Traumatic brain injury (TBI) has long-term impacts on cognition and psychiatric conditions, including depression, anxiety, and aggression. Although there is a noted association between emotional effects and physical disability resulting from TBI, it has been shown that neuropsychiatric symptoms, including memory and cognitive impairment, anxiety, depression, social withdrawal, and aggression, can continue substantially after the initial brain injury, potentially persisting for decades [1]. These symptoms can hinder the rehabilitation process and resumption of employment and result in heavy reliance on the health care system [1]. These related psychiatric symptoms do not have a correlation with the severity of the initial injury or with pain [1].

It has been well demonstrated that TBI is associated with the development of a wide range of neuropsychiatric diseases [2,3]. A likely reason for the increased presence of neuropsychiatric diseases after TBI involves the general mechanisms governing these diseases and their relationship with chronic blood–brain barrier (BBB) permeability and the effects of chronic glutamate neurotoxicity [3]. Depression is a common psychiatric condition in patients with TBI. A recently published, large meta-analysis, consisting of 82 studies in Europe, the United States, Canada, Australia, and New Zealand with 392,834 patients, showed that the odds ratio for depression associated with mild TBI was 3.29 when compared to those without a history of mild TBI [4]. However, previous studies reported that depression after TBI occurred in 35% of cases, with a wide range from 10 to 77% [5,6,7]. This variability may be due to differences among the various populations included, the severity of TBI, the time since injury, and the diverse diagnostic tools utilized [8].

**Figure 1:**
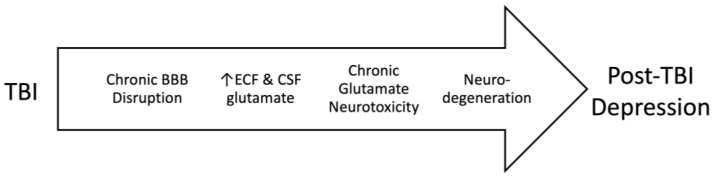
The relationship between TBI, disruption in the blood–brain barrier, chronic glutamate neurotoxicity, and depression.

A relationship between depression and glutamate has been noted definitively for several decades [3]. Our previous work examined the impact of high blood glutamate levels on the development of post-traumatic depression, which was initiated by pathological increases in brain glutamate levels resulting from chronic BBB destruction (Figure 1). In this article, we aim to evaluate the involvement of dietary glutamate in the development of post-traumatic brain injury depression. We hypothesize that glutamate in diet can have an impact on blood glutamate levels and, therefore, depression following TBI. Furthermore, we examine the development of post-TBI depression in Asian countries that traditionally use a high-glutamate diet with the potential to increase blood glutamate levels, compared to countries that incorporate a diet without high glutamate levels, such as Europe, North America, Australia, and New Zealand.

## 2. Materials and Methods

### 2.1. Literature Search

We enacted a literature search to find studies that researched the effects of dietary glutamate in the context of chronic BBB destruction after TBI (Supplementary File S1). On 4 July 2023, we performed a wide search across many databases: MEDLINE (Ovid), Embase (Ovid), PsycINFO (Ovid), Web of Science Core Collection (Clarivate), Scopus (Elsevier), Cumulative Index to Nursing and Allied Health Literature (CINAHL) (EBSCOhost), and the Cochrane Library (Wiley).

### 2.2. Inclusion and Exclusion Criteria

We included publications on the effects of a glutamate-rich diet, as traditional in Asian populations, compared with European and American diets poor in glutamate in the context of chronic BBB destruction after TBI. We used data from recent publications and meta-analyses to compare populations in Europe, the US, Canada, Australia, and New Zealand, with a total of 279,772 cases outside of Asia. The Asian sample included 2,285,938 cases from China, India, Japan, South Korea, Malaysia, Pakistan, and Taiwan (Supplementary Files S1 and S2).

## 3. Results

Our calculated odds ratio (OR) in the Asian group is 1.56 (95% CI 1.532–1.583; Z = 53.13; *p* < 0.001) compared to the European, American, Canadian, Australian, and New Zealand populations during the first year after TBI, which supports our assumption that the prevalence of depression after TBI is higher in Asian countries (see Supplementary Materials). As we noted above, there are significant differences in the assessment and diagnosis of depression in non-Asian countries compared to Asian countries, but we do not think they can fully explain the large differences in the prevalence of depression after TBI.

## 4. Discussion

### 4.1. Pathophysiology of Depression

Depression is one of the most widely observed neuropsychiatric consequences known to occur after TBI. A relationship between depression and glutamate has been confidently noted since the turn of the century [3]. A recent meta-analysis identified that increased blood glutamate levels are strongly correlated with the development of depression [9]. Glutamate is the most plentiful free amino acid in the brain, with concentrations in the plasma at 50–100 μM/L and in the whole brain at 150–300 μM/L. In the whole brain, the concentrations are 10,000–12,000 μM/kg, but only 1–10 μM/L in extracellular fluids. The facilitative and active transport mechanisms of the blood–brain barrier control the gradient between brain cells, blood, and extracellular fluids. A healthy BBB successfully impedes glutamate from traveling between the intraparenchymal and blood compartments. Several mechanisms evoke an increase in brain glutamate that is correlated with TBI (neuronal death, inflammation, impaired glutamatergic recycling and signaling, prolonged stress, astrocytic release of adenosine triphosphate, and other causes of elevated intraparenchymal glutamate), but we believe that the degree of the integrity of the BBB is the fundamental component that mediates the range of glutamate concentration in both healthy and damaged brains [3].

Many variables are implicated in the pathophysiology of depression following TBI, including neuroinflammation, neuroendocrine dysregulation, metabolic abnormalities, neurotransmitter and circuitry dysfunction, neurodegeneration, cell death, axonal injury, maladaptive neuroplasticity, glymphatic system disruption, and long-term disruption in the BBB. These mechanisms may integrate with each other and induce depression after TBI [10]. However, the foundational mechanisms and factors of post-TBI depression remain poorly understood and relatively understudied. Therefore, although there is a relatively high prevalence and severity of depression after TBI, existing treatment options are not completely reliable, as its neurobiological mechanisms are still somewhat unknown. More research studies and a better understanding of the mechanisms that govern post-TBI depression will be extremely beneficial to shape guidelines, protective strategies, and treatment modalities for this multi-faceted neuropsychiatric condition.

Theories about depression originate with several mechanisms, including dysregulation of serotonin and the involvement of the hypothalamic–pituitary–adrenal (HPA) axis, as well as glutamate. Deregulation of the serotonin system is considered the main hypothesis for the development of depression. Pre-clinical and clinical studies have shown an association between major depressive disorder (MDD) and impaired serotonergic transmission as seen by decreases in serotonin neurons and their projections and increases in receptor autoinhibition [11,12]. Impaired responses to antidepressants have also been observed [11,12].

Selective serotonin reuptake inhibitors (SSRIs) are noted as the primary approach for depression according to clinical guidelines concerning TBI treatment. SSRIs raise the levels of serotonin attached to serotonergic postsynaptic receptors, which enables the mediation of several neuropsychiatric conditions including depression, obsessive compulsive disorder, and panic disorder. In the past few decades, some guidelines have suggested that SSRIs are an appropriate therapy for TBI-related depression. However, due to the paucity of robust studies and randomized clinical trials, guidelines for routine use could not be established. More recently, a number of studies have appeared that examined the efficacy of the SSRI sertraline for depression after TBI. These studies have shown that treatment with serotonin-based medications is not effective in restoring cognitive function after TBI, nor have any improvements been reported for post-traumatic stress disorder and post-traumatic anxiety. There remain differing opinions and no clear consensus on the effectiveness of SSRIs and other antidepressants in improving symptoms of post-traumatic depression [13,14,15]. Kreitzer et al. reviewed 1020 articles published prior to September 2017 and did not observe any benefit of antidepressant therapies compared to placebo in treating MDD after TBI [16].

The HPA axis, because of its main contributions to the neuroendocrine system, also plays a critical role in the regulation of responses to stress [17]. The HPA axis mediates stimulations and feedback between the hypothalamus, pituitary, and adrenal glands, which assist in regulating glucocorticoid homeostasis [18]. After encountering a stress stimulus, the HPA axis is engaged through the release of corticotropin-releasing hormone and arginine vasopressin from the hypothalamus. The two hormones induce the pituitary gland to release adrenocorticotropic hormone into the bloodstream, which then causes cortisol production from the adrenal cortex [19]. At the same time, cortisol maintains its physiological function by providing negative feedback to the hypothalamus and pituitary, leading to a reduction in the release of corticotropin-releasing hormone and adrenocorticotropic hormone [20]. In depression, the functionality of this negative feedback loop is debilitated, causing hyperactivity of the HPA axis with higher cortisol levels. An increased level of basal cortisol has been observed to be predictive of depressive episodes [18,19,21,22].

### 4.2. Glutamatergic Hypothesis of Depression

Glutamate participates in most of the fast excitatory transmissions in the brain, and another amino acid neurotransmitter, γ-aminobutyric acid (GABA), impacts most inhibitory transmissions. Glutamate neurons and synapses comprise the largest neurotransmitter system in the brain, being second only to the GABAergic system [3]. Animal and human studies have demonstrated that the glutamate system is a contributing factor in the pathophysiology of depression [3]. Data have demonstrated increased glutamate levels in the blood, cerebrospinal fluid (CSF), and brains of patients with MDD, with evidence showing that antidepressants lower those levels [3]. Elevated glutamate levels have been noted in the frontal cortex as well based on postmortem samples of brain, CSF, and blood plasma of patients with MDD. Plasma glutamate levels, along with alanine and L-serine levels, indicate the disease’s severity [3]. The results of a meta-analysis of magnetic resonance spectroscopy additionally support the theory that glutamatergic neurotransmission plays a role in the pathophysiology of depression [23]. Studies have identified the relationship between dysregulated glutamate receptors and subtypes in postmortem brain samples of patients with MDD and depression [24]. Another meta-analysis by Luykx et al. [25] similarly observed region- and state-specific changes in depression related to concentrations of glutamate and glutamine, an essential amino acid that develops from glutamate conversion. Cumulatively, this evidence implies that disruptions to glutamate receptors and altered blood, plasma, and brain glutamate levels have an impact on the pathophysiology of depression. Research into depressive-like behavior in genetic models of impaired glutamate function and treatment of depression with glutamate-based antidepressants bolster this hypothesis [3].

Among the many factors that have an impact on the development of depression, heightened inflammatory activation of the immune system affects the periphery as well as the central nervous system (CNS) and, therefore, has a strong correlation with the condition. This is additionally evidenced by the strong correlated relationship between diseases with immune activation and symptoms of autoimmune disorders, such as multiple sclerosis, and immune system activation during infections, like sepsis [26]. Similarly, inflammatory responses to TBI have been reviewed in depth [27]. Chronic/dysregulated inflammatory signaling is one proposed pathway leading to impairment and neural circuit dysregulation following TBI as well as mood and anxiety disorders [28]. Neuroinflammation caused by TBI may increase the likelihood of neuropsychiatric disorders directly through chronic inflammatory signaling following TBI or indirectly by inducing the neural immune system to overreact to homeostatic imbalance. However, anti-inflammatory treatments have shown limited efficacy in enhancing long-term TBI outcomes assessed based on mortality, cognitive function, and psychological symptoms [28]. There is much putative evidence that chronic inflammation after TBI increases the likelihood of mood and anxiety disorders, though it remains undetermined if inflammation is a primary factor or only a secondary indicator of the underlying pathology [28].

It has been acknowledged that TBI may have a correlation with related development of chronic neurodegenerative disorders, including mood illnesses [29,30]. A previous diagnosis of TBI has been indicated as a factor in the increased prevalence of Alzheimer’s disease and similar diseases, including early-onset dementias [31].

TBI has a complex pathobiological structure and, therefore, is a complicated neurological condition. The etiology of neurodegeneration has been firmly established as relating to β-amyloid, hyperphosphorylated tau deposition, and neurofibrillary tangles. Because TBI can induce a rapid elevation of these biomarkers, even at younger ages, it has led to a proposed pathobiological linkage between TBI and neurodegeneration [31]. Neuroimaging studies have identified atrophy of frontal and temporal connections in TBI patients that is separate from the acute phase of injury [30]. In the first through fourth years after injury, moderate-to-severe TBI patients have more diffuse white matter atrophy in comparison to control patients of the same age [32]. Animal studies have indicated that TBI can lead to continuing neurodegeneration in addition to associated cognitive or behavioral changes [31].

Until recently, it was unclear if the BBB was severely physically damaged after brain injury over the long term. Recent data using rodent models have shown that restoration of BBB integrity can occur only after 1–3 months or as long as 10 months after injury; restoration of BBB integrity in humans may take years [3]. The BBB is vitally important in preserving CNS homeostasis by limiting the movement of harmful substances from the circulating bloodstream into the brain parenchyma. BBB destruction can prompt and cause worsening of depression in several ways. Poor endothelial function and BBB breakdown cause cerebral perfusion impairments, thereby causing brain damage and compromised emotional and cognitive functions [18].

Other neurobiological factors, such as elevated levels of biomarkers after TBI associated with neurodegeneration, the flushing out of potentially toxic metabolites via the glymphatic system, neuroendocrine dysregulation, metabolic abnormalities, stress, and neurotransmitter and circuitry dysfunction [10,29,33], may also contribute to the development of post-TBI depression.

### 4.3. Glutamate Neurotoxicity

Glutamate neurotoxicity describes neurotoxicity resulting from excessive glutamate that causes neuronal degeneration and dysfunction [34,35,36,37]. Based on this relationship, recent studies on mood disorders have specifically addressed the glutamatergic system as a site for new therapeutic modalities for antidepressants and other methods [3,38,39]. A healthy BBB has been shown to effectively protect the brain from high glutamate levels in the blood. Also, the concentration of glutamate in the CSF is correlated with the concentration of glutamate in the blood after BBB disruption. Because CSF glutamate concentration is based on the level of BBB disruption, BBB permeability is an essential aspect in maintaining healthy levels of glutamate in the brain [40].

After acute brain injuries (including cerebral ischemia, hypoglycemia, ischemic strokes, and TBI), excitotoxicity may arise due to elevated extracellular glutamate levels, leading to the overactivation of ionotropic glutamate receptors [3,40].

It has also been proposed that chronic glutamate neurotoxicity occurs in some neurodegenerative diseases, including amyotrophic lateral sclerosis, Alzheimer’s disease, and Huntington’s disease [3]. For these neurogenerative diseases, research suggests that chronic excitotoxicity takes place as a result of cellular death occurring over a longer period of time when neurons encounter increased glutamate, leading to a gradual progression toward cell death [3]. This understanding of the development of neurodegeneration due to glutamate neurotoxicity assists in providing new options for treating acute and chronic neurological conditions revolving around the glutamatergic system.

### 4.4. Diet as a Factor in Blood Glutamate Concentration

Blood glutamate concentrations are generally stable under physiological conditions, but a number of factors can influence the increase or decrease in its concentration. As suggested by the articles reviewed herein, dietary intake can have an impact on blood glutamate concentrations due to the presence of glutamate in many foods. Asian countries traditionally have a glutamate-rich diet and account for about 88% of global consumption of glutamate [41] and 94% of the world’s monosodium glutamate (MSG) production capacity [42]. MSG is a common flavor enhancer that is used in many cuisines around the world. It is particularly popular in East and Southeast Asian cooking, where it is often used in dishes such as soups, stir-fries, and marinades. Several pharmacokinetic studies have shed light on the dynamics of blood glutamate levels based on dietary intake. Rutten et al. administered oral glutamate doses of 30 mg per kilogram of body weight every 20 min for 220 min. They found that plasma glutamate levels peaked after 80 min at five times the basal value (601 ± 68 μmol/L). Further administration of the drug did not significantly increase glutamate levels, suggesting a saturable level of intestinal absorption and clearance [43].

MSG intake of 16.0 mg/kg of body weight is considered safe [42]. The average daily consumption of MSG is around 0.3–0.5 g/day in European countries and the United States [42] and 1.2–4 g/day in Asian countries [44]. However, its neurotoxicity in light of the chronic impairment associated with BBB permeability is of concern. Stegink et al. [45] conducted a study in which healthy subjects were given beef consommé containing 1 mg of glutamate per kilogram of body weight, alone or with supplemental MSG at doses of 25 or 50 mg/kg body weight. They observed a dose-dependent increase in plasma glutamate, which peaked at 30 min.

In studies involving the administration of 150 mg MSG/kg body weight as a bolus to healthy adults in the post-absorption state, plasma glutamate levels reached their peak after 45 min, exceeding the basal value by 19 times (594 ± 465 μmol/L), and returned to baseline at 180 min after administration [46]. Similar results were obtained by Fernstrom et al., who administered 160 mg MSG/kg body weight to healthy adults in the post-absorption state. They observed a peak in plasma glutamate levels (at 11 times the basal value [530 μmol/L]) after 60 min; by 180 min, the levels had returned to baseline [47]. Graham et al. conducted a study in which healthy young adults were orally administered 150 mg MSG/kg body weight, which resulted in peak plasma glutamate levels (8 times the basal value [437 μmol/L]) 30 or 45 min after administration. After 90 min, glutamate levels were no longer significantly elevated compared to baseline levels [48].

In addition, studies have noted the relevance of food intake on plasma glutamate levels. Tsai and Huang reported that after lunch and dinner, plasma glutamate levels increased 1.4-fold from baseline, with peak concentrations at about 60 min after a meal [49]. Stegink et al. [50] observed a similar magnitude of increase in plasma glutamate after healthy adults consumed a cooked liquid meal containing 55 mg of protein-bound glutamate per kilogram of body weight, reaching a 1.6-fold increase over baseline with a peak after 45 min. When the food contained 169 mg of protein-bound glutamate per kilogram of body weight, plasma glutamate concentrations increased 2.3-fold over baseline after 120–150 min.

Thus, we can conclude that the use of MSG results in a rapid increase in plasma glutamate levels, with a peak concentration of approximately 600–700 μmol/L at 30–60 min after ingestion, and the concentration then decreases to baseline within 90–180 min [51].

Although the Food and Drug Administration (FDA) has deemed MSG safe for consumption, several animal studies have pointed to the potential negative effects associated with chronic MSG consumption. These adverse effects have been observed in different organs, such as the thymus, brain, pancreas, testis, liver, and kidney, and have been associated with several diseases, including obesity, hypertension, and headaches, as well as with asthma exacerbation and reproductive conditions [52].

Because glutamate plays an important role as an excitatory neurotransmitter in the CNS, when it reaches excessive levels, the resultant excitotoxicity can lead to severe neuronal damage and cognitive and behavioral impairments. In one study on neonatal rats, overactivation of glutamate receptors was induced by MSG solution administration and resulted in irregularities in all amino acids, leading to behavioral changes such as screeching, tail stiffness, head nodding, and generalized convulsions or seizure-like conditions [53]. Another neonatal rat model showed excitotoxicity of glutamate, with cellular degeneration of 11.5% in the hippocampus of rats given MSG compared to rats in the control group [54]. Negative changes to dendritic arborization and dendritic spine density were also attributable to the cyto-excitotoxic effect of MSG, which leads to impairments in the hippocampus. In another study by Dief et al. [55], male Wistar rats that were administered MSG showed a reduction in cyclic-AMPK level in the hippocampus by 43% with oral MSG administration and by 31% with subcutaneous MSG administration compared to rats not given MSG. The MSG group also showed a two-fold increase in the Fas ligand, a substance involved in cell death [42]. One study placed MSG in drinking water that was available ad libitum to female rats in the last two weeks of pregnancy, and the rats born from the MSG group had reduced birth weight, increased weight at day 28 of life, decreased open-field activity and less lactation at day 35 of life, and made more maze errors at day 60 of life [56,57]. The neurotoxic effects of glutamate were also observed in another study in the cerebellar cortex of male albino rats receiving 3 g/kg/day [58].

Recent studies have revealed an intriguing interaction between the gut microbiota and the brain neurotransmitters dopamine, serotonin, GABA, and glutamate [59]. These interactions provide new insights into the possible relationship between dietary factors, neurotransmitter functions, and psychiatric disorders such as depression. Some researchers have suggested that diets high in sodium glutamate may increase blood levels of glutamate and glutamic acid, leading to hyperglutamatergic neurotransmission. Hyperglutamatergic neurotransmission has been associated with several different psychiatric conditions, including depression [59]. Studies have shown that consuming a diet rich in sodium glutamate can lead to depressive behaviors in rodents, including decreased social interaction, anhedonia, and behavioral despair [59,60,61]. Preclinical studies have revealed a potential link between sodium glutamate intake and the development of anxious and depressive-like phenotypes [59]. Animal models receiving sodium glutamate showed increased anxiety and behavioral despair [62,63,64,65,66]. The behavior of rats suffering from post-traumatic depression and anxiety and receiving treatment aimed at reducing blood and CSF glutamate levels did not differ from that of naïve rats after the completion of treatment [67,68]. These data suggest that there is a very close relationship between dietary glutamate consumption and the development of depression.

### 4.5. Other Factors

In this review, we examined the effects of dietary glutamate in the context of BBB destruction following TBI as a trigger for the initiation of degenerative processes that can eventually lead to depression. However, there are many other factors besides glutamate that contribute to depression, including socioeconomic factors, gender, stress levels, chronic infections, and chronic illnesses. Also, in addition to dietary intake, other factors influence changes in blood glutamate levels, such as injury in the spinal cord and blood cell disruption [69,70]. In vitro studies have observed changes in the endothelial barrier due to exposure to soluble polymorphonuclear leukocyte-derived glutamate during inflammatory states [71]. Another possible pathway of glutamate is bone. In one study, osteoclasts were identified as secreting glutamate when stimulated with KCl or adenosine triphosphate [72]. The effects of stress [73], circadian rhythms [49], gender [74,75], age [76], and pain [77] on blood or plasma glutamate concentrations have also been documented.

### 4.6. Potential Treatment Strategies

**Figure 2:**
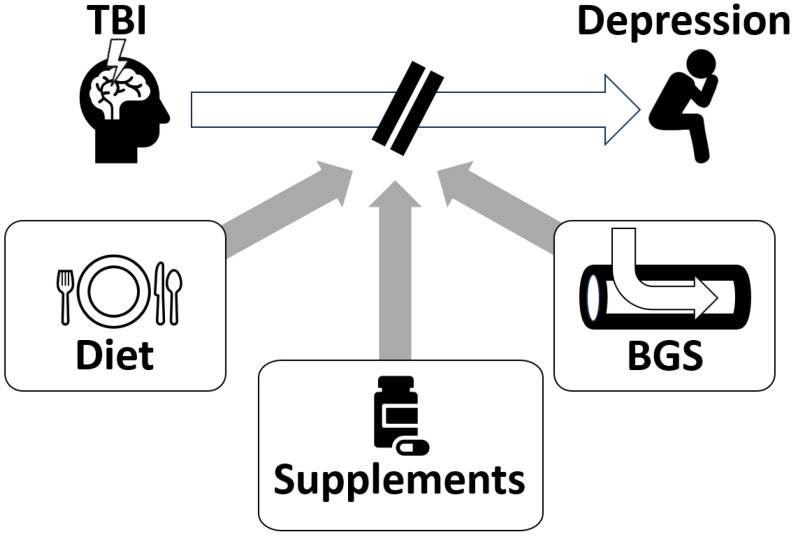
Potential treatment strategies for post-TBI depression include dietary glutamate modulation, dietary supplements, and blood glutamate scavenging (BGS). Based on the role of glutamate in the pathophysiology of this condition, we hypothesize that these three approaches may be beneficial in alleviating depression after TBI.

Based on the theory of blood glutamate reduction for alleviating depression after TBI, we propose several potential treatment strategies (Figure 2).

#### 4.6.1. Diet

In this review, we examined the role of a glutamate-rich diet in the development of depression. Although a glutamate-rich diet is not neurotoxic when the blood–brain barrier is healthy, there is strong evidence that when the barrier is compromised, chronic glutamate neurotoxicity can be a trigger for the development of neurodegeneration and subsequent depression. Based on this hypothesis, we can assume that a glutamate-poor diet can be considered as a potential method for the prevention of neurodegenerative processes and modulation of glutamate levels in patients’ diet can contribute to the treatment and prevention of depression.

#### 4.6.2. Food Supplements

Dietary supplements have gained popularity in recent years as people look for effective yet simple and non-invasive strategies to improve health. Pyruvate serves as an important energy intermediate, as a critical component in the production of ATP, and as a basic energy currency of cells, and it has been approved as a dietary supplement. Oxaloacetate, an intermediate product of the citric acid cycle, is also a dietary supplement and has a significant impact on various metabolic pathways, including gluconeogenesis, the urea cycle, amino acid synthesis, and fatty acid synthesis. Both of these substances can effectively reduce glutamate levels by breaking it down to alpha-ketoglutarate aspartate and alanine. Pyruvate and oxaloacetate are FDA-approved dietary supplements as over-the-counter drugs.

#### 4.6.3. Blood Glutamate Scavenging

Excessively high levels of brain glutamate can also be mitigated by manipulating the brain–blood glutamate equilibrium and inducing excess glutamate from the brain’s interstitial fluid to flow into the body’s circulatory system [3]. Glutamate transporters on the endothelial cells of brain capillaries allow the extraction of glutamate from interstitial fluid [3]. This process of lowering the level of glutamate is known as blood glutamate scavenging. Blood glutamate scavenging is able to accomplish this without compromising mechanisms of learning since it does not involve the impediment or direct stimulation of synaptic glutamate receptors. Treatments that have been proven in a rat model to reduce the first neuroanatomical and neurological symptoms of TBI involves the administration of the enzymes glutamic oxaloacetic transaminase and serum glutamic pyruvic transaminase, and their co-substrates oxaloacetic acid and pyruvate [3].

## 5. Conclusions

High blood glutamate levels are strongly correlated with the development of post-traumatic brain injury depression. We theorize that BBB permeability is a determining component in the onset of neurodegeneration processes through glutamate neurotoxicity and the subsequent appearance of neuropsychiatric pathology. Therefore, methods of blood glutamate level reduction and restoration of BBB integrity may be effective tools for the treatment and prevention of post-TBI depression. Blood glutamate levels can be affected by a number of factors, both internal and external, that can cause significant increases, while a healthy BBB is able to successfully shield the brain from the neurotoxic effects of blood glutamate. Food intake of glutamate appears to be the most significant out of all the easily controllable factors that can increase blood glutamate levels. In chronic disorders involving BBB disruption following TBI, chronically high blood glutamate concentrations resulting from a glutamate-rich diet can be neurotoxic to the brain and initiate neurodegeneration processes that will eventually lead to depression. A low-glutamate diet, medications that target the glutamate system, and food supplements that reduce blood glutamate levels may be effective strategies for treating post-TBI depression.

## Data Availability

Not applicable.

## Supplementary Materials

The following supporting information can be downloaded at https://www.mdpi.com/article/10.3390/nu15214681/s1. File S1: Literature search results; File S2: Prevalence of post-TBI depression in Asian countries versus control group or indigenous population [78,79,80,81,82,83,84,85,86,87,88,89,90,91,92,93,94,95,96,97,98,99,100,101,102,103,104,105,106,107,108,109,110,111,112,113,114,115,116,117,118,119,120,121,122,123,124,125,126,127,128,129,130,131,132,133,134,135,136,137,138,139,140,141,142,143,144,145,146,147,148,149,150,151,152,153,154,155,156,157,158,159,160,161,162,163,164,165,166,167,168,169,170,171,172,173,174,175,176,177,178,179,180,181,182,183,184,185].
