# Supplementary material for: Diet’s Impact on Post-Traumatic Brain Injury Depression: Exploring Neurodegeneration, Chronic Blood–Brain Barrier Destruction, and Glutamate Neurotoxicity Mechanisms
Source: Nutrients. 2023 Nov 4;15(21):4681. doi: 10.3390/nu15214681 (PMC10649677; doi:10.3390/nu15214681)
Supplement: Supplementary file 1 [file nutrients-15-04681-s001.zip › File S1.pdf]

Study	Population	Participant (n)		Odds Ratio	95% CI	Reference
		TBI	Control			
Dean, et al. 2013	US	36	36	0.23	0.09-0.52	[4,78]
Losoi, et al. 2016	US	69	37	0.40	0.11-1.41	[4,79]
Amick, et al. 2018	US	22662	26153	0.61	0.58-0.65	[4,80]
Bryant, et al. 2010	Australia	321	321	0.67	0.45-0.99	[4,81]
Ponsford J, et al. 2019	Australia	343	343	1.04	0.481-2.079	[82]
Pogoda, et al. 2012	US	9998	3748	1.04	0.96-1.13	[4,83]
Dretsch, et al. 2015	US	167	291	1.26	0.88-1.79	[4,84]
Waljas M, et al. 2015	Europe	103	36	1.27	0.64-2.52	[4,85]
Leveille, et al. 2017	Canada	22	28	1.32	0.48-3.67	[4,86]
Tremblay, et al. 2013	Canada	15	15	1.32	0.36-4.82	[4,87]
Walker, et al. 2017	US	176	40	1.35	0.72-2.53	[4,88]
LaFrance, et al. 2013	US	41	51	1.35	0.06–1.19	[4,89]
Verfaellie, et al. 2014	US	53	39	1.45	0.69-3.05	[4,90]
Vasterling, et al. 2000	US	68	692	1.49	0.80-2.79	[4,91]
Nordhaug et al. 2018	Europe	294	25662	1.49	0.20-1.85	[4,92]
Barnes, et al. 2012	US	46	46	1.52	0.72-3.21	[4,93]
Palombo, et al. 2015	US	50	25	1.57	0.56-4.43	[4,94]
Spira, et al. 2014	US	98	305	1.62	1.07-2.44	[4,95]
Decq et al. 2016	Europe	217	158	1.63	1.12-2.37	[4,96]
Mickeviciene et al. 2002	Europe	131	146	1.65	0.99-2.74	[4,97]
Jurick, et al. 2018	US	42	28	1.65	0.70-3.91	[4,98]
MacGregor 2013	US	334	658	1.72	1.21-2.44	[4,99]
Polusny, et al. 2011	US	60	748	1.90	1.18-3.04	[4,100]
Ozen, et al.2010	Canada	43	44	1.97	0.92-4.24	[4,101]
Baldassrre, et al. 2015	US	188	210	2.03	1.30-3.19	[4,102]
Tarazi, et al. 2018	Canada	45	25	2.03	0.83-5.01	[4,103]
Callahan, et al. 2018	US	42	36	2.08	0.91-4.73	[4,104]
Lee, et al. 2015	Canada	182	2843	2.10	1.56-2.81	[4,105]
Dismuke-Greer, et al. 2018	US	300	56	2.12	1.13-3.96	[4,106]
Barker-Collo, et al. 2018	New Zealand	341	341	2.17	1.051-4.125	[107]
Vasterling, et al. 2012	US	87	84	2.25	1.30-3.89	[4,108]
Gardner, et al. 2017	Australia	16	16	2.29	0.64-8.20	[4,109]
Hoot, et al. 2018	US	376	73	2.32	1.31-4.09	[4,110]
Gill, et al. 2018	US	42	22	2.34	0.91-5.99	[4,111]
Donnelly, et al. 2018	US	130	225	2.59	1.68-3.98	[4,112]
Lippa, et al. 2015	US	99	156	2.64	1.52-4.57	[4,113]
Johansson et al. 2009	Europe	46	40	2.66	0.1-67.62	[4,114]
Albrecht, Jennifer, et al. 2020	US	78044	76107	2.81	2.725-2.906	[115]
Vanderploeg, et al. 2007	US	254	3214	3.00	2.03-4.45	[4,116]
Olivera, et al. 2015	US	70	28	3.10	1.39-6.91	[4,117]
Morissette, et al. 2011	US	98	115	3.13	1.88-5.20	[4,118]
Bell, et al. 1999	US	20	20	3.25	1.02-10.34	[4,119]
Rogers, et al. 2015	Australia	10	10	3.29	0.63-17.05	[4,120]
Yvette Always, et al. 2012	New Zealand	43	43	3.52	0.494-14.738	[121]
Alice Theadom, et al. 2016	New Zealand	341	341	3.58	1.812-6.560	[122]
S Barker-Collo, et al. 2015	New Zealand	315	315	3.68	1.831-6.953	[123]

Schoenhuber, et al.1988	Europe	35	35	3.63	1.5-8.78	[4,124]
Kerr, et al. 2018	US	172	32	4.10	0.94-17.81	[4,125]
Carrier, et al. 2018	Canada	43	40	4.10	1.80-9.33	[126]
Mac Donald, et al. 2017	US	50	44	4.22	1.52-11.70	[4,127]
Hoge, et al. 2008	US	368	1673	4.39	2.91-6.63	[4,128]
Gaines, et al. 2016	US	57	57	4.39	2.17-8.90	[4,129]
A J Osborn, et al. 2017	Australia	353	353	4.41	2.512-8.141	[130]
Biyao Wang, et al. 2021	Europe	1683	1683	4.84	3.660-6.507	[131]
Mac Donald, et al. 2015	US	38	34	4.95	0.99-24.71	[4,132]
Iverson, et al. 2015	US	33	119	5.00	2.38-10.54	[4,133]
Kerr et al. 2012	US	679	365	5.21	2.78-9.75	[4,134]
Graham, et al. 2013	US	41	26	5.26	2.05-13.47	[4,135]
Wilk, et al. 2012	US	260	846	5.42	3.39-8.67	[4,136]
Murray B. Stein, et al. 2019	US	1155	1155	5.45	4.036-7.465	[137]
Didehbani, et al. 2013	US	30	29	5.75	1.43-23.14	[4,138]
Ashley Di Battista, et al. 2014	New Zealand	20	20	5.79	0.481-46.91	[139]
Strigo, et al. 2018	US	20	24	5.81	1.83-18.47	[4,140]
Petrie, et al. 2014	US	34	17	5.93	1.17-30.17	[4,141]
Bomyea, et al. 2016	US	52	32	6.11	2.58-14.47	[4,142]
Baker, et al. 2018	US	21	21	6.23	0.67-58.23	[4,143]
Coughlin, et al. 2017	US	14	16	6.62	0.29-149.36	[4,144]
Epstein, et al. 2016	US	55	27	7.46	3.03-18.39	[4,145]
Konrad et al. 2011	Europe	33	33	7.69	0.38-154.38	[4,146]
Schiehser, et al. 2017	US	60	40	8.67	3.88-19.37	[4,147]
Whelan-Goodinson, et al.2009	New Zealand	100	100	9.14	2.820 -25.22	[148]
Peskind, et al. 2011	US	12	12	9.21	0.42-199.77	[4,149]
Drapeau, et al. 2017	Canada	20	11	9.39	2.16-40.85	[4,150]
Dean P McKenzie, et al. 2018	New Zealand	138	138	9.53	3.698-25.681	[151]
Jane Dahm 2013	New Zealand	123	123	10.79	3.681-25.904	[152]
Dailey, et al. 2018	US	15	14	10.80	2.44-47.92	[4,153]
Guskiewicz, et al. 2007	US	1513	1039	11.25	6.89-18.36	[4,154]
Astafiev, et al. 2016	US	20	22	12.30	0.63-242.05	[4,155]
Chong 2018	US	33	33	13.60	4.91-37.68	[4,156]
Raikes, et al. 2018	US	5	18	14.01	1.83-107.59	[4,157]
Pineau, et al. 2015	Canada	25	25	15.33	4.73-49.70	[4,158]
Newberg, et al. 2014	US	25	10	16.61	0.88-314.17	[4,159]
Donnell, et al. 2012	US	154	3001	17.12	10.90-26.86	[4,160]
Sponheim, et al. 2011	US	9	8	18.73	2.39-146.64	[4,161]
Maruta et al. 2016	US	33	140	27.11	11.90-71.26	[4,162]
Morey et al. 2013	US	30	70	38.09	14.02-103.50	[4,163]
Himanen et al. 2009	Europe	17	31	70.11	3.71-1326	[4,164]
Suhr and Gunstad, et al. 2002	US	63	50	104.6	6.22-1758.73	[4,165]
Walker et al. 2013	US	29	58	372.4	96.32-1440	[4,166]
Small et al. 2013	US	5	5	512.9	10.58-24853	[4,167]
Raskin, et al. 1997	US	10	10	6701	197-228190	[4,168]
**Total**		**279772**		**2.74237**	**2.671 - 2.815**	**p < 0.001**

Supplement S1. Prevalence of post-TBI depression in Europe, the United States, Canada, Australia, and New Zealand ver. of control group or indigenous population (https://ourworldindata.org/mental-health)**.**

	Event (depression)	No Event
**TBI group**	a	b
**Control group**	c	d

We calculated the confidence Interval and the odds ratio using the following formulas.

- **Odds ratio** = (a*d) / (b*c)
- **Lower 95% CI** = $e^{\ln(OR) - 1.96\sqrt{(1/a + 1/b + 1/c + 1/d)}}$
- **Upper 95% CI** = $e^{\ln(OR) + 1.96\sqrt{(1/a + 1/b + 1/c + 1/d)}}$

Whereas in the publication the data were presented as a percentage of the number of cases, we recalculated and presented the data as an odds ratio.

In the case of publication with only a TBI group without a control group, we used a control group with the same number of cases for that population, and data on the prevalence of depression in that population were taken from https://ourworldindata.org/mental-health for the same time period, a similar method was done in a meta-analysis where a control group with the same number of patients was taken from the National Health Insurance Service-National Health Information Database (NHIS-NHID) in South Korea [169].

Odds ratio was calculated as previously described [170-173].
