# Supplementary material for: Diet’s Impact on Post-Traumatic Brain Injury Depression: Exploring Neurodegeneration, Chronic Blood–Brain Barrier Destruction, and Glutamate Neurotoxicity Mechanisms
Source: Nutrients. 2023 Nov 4;15(21):4681. doi: 10.3390/nu15214681 (PMC10649677; doi:10.3390/nu15214681)
Supplement: Supplementary file 1 [file nutrients-15-04681-s001.zip › File S2.pdf]

| Study | Population | Participant (n) | | Odds Ratio | 95% CI | Reference |
|---|---|---|---|---|---|---|
| | | TBI | Control | | | |
| Li, Gangqin, et al. 2022 | China | 72 | 72 | 41.36 | 9.42-181.72 | [174] |
| Tomar, Sachin, et al. 2018 | India | 100 | 100 | 126 | 40.54-391.66 | [175] |
| Ansari, Ahmed, et al. 2014 | India | 250 | 250 | 14.8 | 7.25-30.23 | [176] |
| Jain, Akhilesh, et al. 2014 | India | 204 | 204 | 11.98 | 5.58-25.75 | [177] |
| Devi, Yashodha, et al. 2020 | India | 50 | 50 | 7.58 | 1.6-35.93 | [178] |
| Chaurasiya, Akanksha, et al. 2021-a | India | 39 | 39 | 21.28 | 2.63-172.15 | [179] |
| Chaurasiya, Akanksha, et al. 2021-b | India | 39 | 39 | 14.93 | 1.82-122.46 | [180] |
| Ubukata, Shiho, et al. 2022 | Japan | 88 | 88 | 83.13 | 19.12-361.42 | [181] |
| Jang, Sung Ho, et al. 2020 | Korea | 58 | 58 | 51 | 6.54-397.97 | [182] |
| Choi, Yoonjeong, et al. 2022 | Korea | 1141593 | 1141593 | 1.7 | 1.69-1.71 | [169] |
| Looi, Mun Choon, et al. 2023 | Malaysia | 309 | 309 | 9.24 | 4.93-17.33 | [183] |
| Al-Kader, Dania A., et al. 2022 | Pakistan | 31 | 31 | 4.16 | 1.39-12.5 | [184] |
| Lin, Mau-Roung, et al. 2010 | Taiwan | 146 | 146 | 24.24 | 8.51-69.08 | [185] |
| **Total** | | **2285938** | | **2.704** | **1.692-1.715** | **p < 0.001** |

**Supplement S2. Prevalence of post-TBI depression in Asian countries versus control group or indigenous population.** (https://ourworldindata.org/mental-health).

| | Event (depression) | No Event |
|---|---|---|
| **TBI group** | a | b |
| **Control group** | c | d |

We calculated the confidence Interval and the odds ratio using the following formulas.

- **Odds ratio** = (a*d) / (b*c)
- **Lower 95% CI** = $e^{\ln(OR) - 1.96\sqrt{(1/a + 1/b + 1/c + 1/d)}}$
- **Upper 95% CI** = $e^{\ln(OR) + 1.96\sqrt{(1/a + 1/b + 1/c + 1/d)}}$

Whereas in the publication the data were presented as a percentage of the number of cases, we recalculated and presented the data as an odds ratio.

In the case of publication with only a TBI group without a control group, we used a control group with the same number of cases for that population, and data on the prevalence of depression in that population were taken from https://ourworldindata.org/mental-health for the same time period, a similar method was done in a meta-analysis where a control group with the same number of patients was taken from the National Health Insurance Service-National Health Information Database (NHIS-NHID) in South Korea [169].

Odds ratio was calculated as previously described [170-173].
